# Experimental and Numerical Study of Viscoelastic Properties of Polymeric Interlayers Used for Laminated Glass: Determination of Material Parameters

**DOI:** 10.3390/ma12142241

**Published:** 2019-07-11

**Authors:** Tomáš Hána, Tomáš Janda, Jaroslav Schmidt, Alena Zemanová, Michal Šejnoha, Martina Eliášová, Miroslav Vokáč

**Affiliations:** 1 Klokner Institute, Research and Experimental Institute of Building Materials and Building Structures, Šolínova 7, 166 08 Prague 6, Czech Republic; 2 Department of Mechanics, Faculty of Civil Engineering, Czech Technical University in Prague, Thákurova 7, 166 29 Prague 6, Czech Republic; 3 Department of Steel and Timber Structures, Faculty of Civil Engineering, Czech Technical University in Prague, Thákurova 7, 166 29 Prague 6, Czech Republic

**Keywords:** laminated glass, polymer, polyvinyl butyral, ethylene-vinyl acetate, generalized Maxwell model, rheometer, dynamic torsional test, dynamic shear test

## Abstract

An accurate material representation of polymeric interlayers in laminated glass panes has proved fundamental for a reliable prediction of their response in both static and dynamic loading regimes. This issue is addressed in the present contribution by examining the time–temperature sensitivity of the shear stiffness of two widely used interlayers made of polyvinyl butyral (TROSIFOL BG R20) and ethylene-vinyl acetate (EVALAM 80-120). To that end, an experimental program has been executed to compare the applicability of two experimental techniques, (i) dynamic torsional tests and (ii) dynamic single-lap shear tests, in providing data needed in a subsequent calibration of a suitable material model. Herein, attention is limited to the identification of material parameters of the generalized Maxwell chain model through the combination of linear regression and the Nelder–Mead method. The choice of the viscoelastic material model has also been supported experimentally. The resulting model parameters confirmed a strong material variability of both interlayers with temperature and time. While higher initial shear stiffness was observed for the polyvinyl butyral interlayer in general, the ethylene-vinyl acetate interlayer exhibited a less pronounced decay of stiffness over time and a stiffer response in long-term loading.

## 1. Introduction

The structural behavior of laminated glass is governed by the material properties and type of its components, basically by a polymeric interlayer keeping the individual glass panes together. This interlayer plays a significant role in a laminated glass panel. It is well known that glass is a brittle material. Before its fracture, the polymeric interlayer provides shear coupling of glass panes and has other additional functions, such as sound and vibration damping, solar and energy control, or an aesthetic function. After the glass fracture, the interlayer keeps the glass fragments together, provides post-breakage resistance, safety for persons, and prevention of injuries, and increases the security of buildings.

The interlayer is made of a polymer such as polyvinyl butyral (PVB), ethylene-vinyl acetate (EVA), or other advanced materials. The performance of these polymers is time- and temperature-dependent. At small strains, the linear viscoelastic behavior is observed [1,2,3]. A critical issue for numerical or analytical modeling of laminated glass elements is the knowledge of their mechanical properties. For most of the interlayers, the data of their thermo-mechanical description are missing in the literature.

Furthermore, producers often do not provide details about the testing procedure or omit the temperature-sensitiveness of the interlayer [4]. Only a few manufacturers provide enhanced mechanical properties of their interlayers [5], thus there is currently no accessible database of interlayers with their thermo-mechanical properties. Taking from the literature mechanical properties determined for a certain type of material, but not directly for the particular interlayer, may lead to an incorrect or unreliable prediction of the laminated glass response [6].

Many types of polymer interlayers are of interest when analyzing post-breaking behavior of laminated glass [7]. The mechanical characteristics of two such interlayers, PVB (TROSIFOL^®^ BG R20) and EVA (EVALAM^®^ 80-120) in particular, are examined in this study. A brief review of current approaches focusing on the modeling of mechanical properties of polymers opens, in Section 2.1, the theoretical part of the paper. The generalized Maxwell model is outlined next in Section 2.2, and the effect of temperature on the stiffness of the interlayer is discussed in Section 2.3. Finally, the formulation of the identification method of the material parameters fitting the experimental data obtained from a cyclic loading is described in Section 2.4. The experimental program is introduced in Section 3, and the experimental data together with the performance of the generalized Maxwell model are presented in Section 4. The main findings and concluding remarks are then summarized in Section 5.

## 2. Material Model

This section reviews the principles of viscoelastic models for polymers and describes the fitting procedure to obtain the mechanical characteristics of polymeric interlayers.

### 2.1. Overview

To capture the viscoelastic time- and temperature-dependent behavior of polymers, a classical rheological model consisting of a few chains or relatively new models based on fractional calculus can be used. The generalized Maxwell model is the most extended approach for modeling the time response of polymeric interlayers in laminated glass elements, e.g., [4,8,9,10,11]. However, this response could be also approximated by the generalized Kelvin–Voigt or Zener models, originally intended for other materials, such as concrete, asphalt, or epoxy matrices in fibrous composites. The fractional version of any conventional viscoelastic model can be developed [12,13]. Their authors point out that fractional derivative models with only a few parameters can accurately describe the response of a polymeric interlayer [14].

In the case of the generalized Maxwell model, the number of chains depends on the frequency range of the studied problem and can be high to minimize the wave-like shape of the fitted curve corresponding to the shear modulus [15]. Despite this drawback, this generalized viscoelastic model is adopted in our study as it is more convenient within finite element simulations, e.g., [16,17,18]. A comparison of the performance of the generalized Maxwell model and a fractional (Zener) model in [19] shows that both models are quantitatively equivalent. Finally, the physical meaning of some fractional derivative models is less intuitive. When applied in the time domain, the fractional operators make the task more complicated, and these methods can be less efficient [20,21].

### 2.2. Generalized Maxwell Model

**Figure 1:**
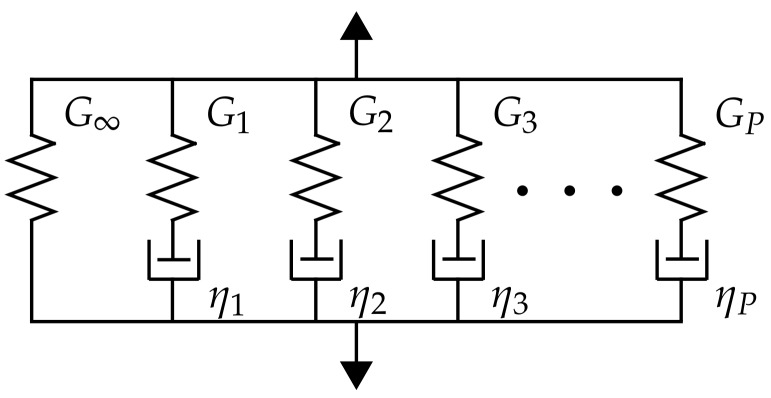
Scheme of the generalized Maxwell chain.

In this section, the viscoelastic constitutive model is briefly recalled. As plotted in Figure 1, the generalized Maxwell model is a parallel chain of a single spring and several spring-dashpot Maxwell units. Indexed by p∈〈1,P〉, the shear stiffness of the spring $G_{p}$ and the viscosity of the purely viscous damper $\eta_{p}$ correspond to the *p*-th Maxwell unit, and the stiffness $G_{\infty }$ characterizes the long-term response of the chain.

For harmonic loading, stresses and strains of a viscoelastic material are related by a complex dynamic shear modulus [22] written as

(1) $$G^{*}\left(\omega \right)=G^{\prime }\left(\omega \right)+iG^{\prime \prime }\left(\omega \right),$$

where ω is a given angular frequency, and $i=\sqrt{-1}$ is the imaginary unit. The real and imaginary part of the complex shear modulus, i.e., the storage modulus $G^{\prime }$ and the loss modulus $G^{\prime \prime }$, respectively, are expressed using the Prony series in the Maxwell model [23] as

(2a) $$\begin{array}{cc} G^{\prime }\left(\omega \right) & =G_{\infty }+\sum_{p=1}^{P}G_{p}\frac{\theta_{p,0}^{2}\omega^{2}}{1+\theta_{p,0}^{2}\omega^{2}}, \end{array}$$

(2b) $$\begin{array}{cc} G^{\prime \prime }\left(\omega \right) & =\sum_{p=1}^{P}G_{p}\frac{\theta_{p,0}\omega }{1+\theta_{p,0}^{2}\omega^{2}}, \end{array}$$

where $\theta_{p,0}$ refers to the relaxation time in [s] of the *p*-th unit for a given temperature $T_{0}$, given by the ratio of the dashpot viscosity and the spring stiffness as

(3) $$\theta_{p,0}=\frac{\eta_{p}}{G_{p}}.$$

In the time domain, the Prony representation of the relaxation shear modulus of the generalized Maxwell model for a given time instant *t* takes the form

(4) $$\begin{array}{c} G\left(t\right)=G_{\infty }+\sum_{p=1}^{P}G_{p}\exp\left(-\frac{t}{\theta_{p,0}}\right). \end{array}$$

### 2.3. Temperature Shifting

Mechanical properties of polymer interlayers show dependence on multiple external effects such as current temperature, experienced thermal cycles, or humidity exposure [24]. The temperature effect in particular is the subject of the present study because it significantly influences the relaxation behavior of the polymer [25]. When the temperature is increased, relaxation times of the polymer decrease accordingly. This correlates with an increase of the load time in static tests or a decrease of the frequency in dynamic tests. This property is taken into account by introducing the shifted relaxation time [26]

(5) $$\theta_{p}\left(T\right)=a_{T}\left(T\right)\theta_{p,0}=a_{T}\left(T\right)\frac{\eta_{p}}{G_{p}},$$

where $a_{T}$ is the temperature shift factor.

Introducing the shifted relaxation time $\theta_{p}\left(T\right)$ in Equations (2a) – (4) in place of $\theta_{p,0}$ thus provides the temperature-dependent moduli in the form

(6a) $$\begin{array}{cc} G^{\prime }\left(\omega ,T\right) & =G_{\infty }+\sum_{p=1}^{P}G_{p}\frac{\theta_{p}^{2}\left(T\right)\omega^{2}}{1+\theta_{p}^{2}\left(T\right)\omega^{2}}, \end{array}$$

(6b) $$\begin{array}{cc} G^{\prime \prime }\left(\omega ,T\right) & =\sum_{p=1}^{P}G_{p}\frac{\theta_{p}\left(T\right)\omega }{1+\theta_{p}^{2}\left(T\right)\omega^{2}}, \end{array}$$

and

(7) $$\begin{array}{c} G\left(t,T\right)=G_{\infty }+\sum_{p=1}^{P}G_{p}\exp\left(-\frac{t}{\theta_{p}\left(T\right)}\right). \end{array}$$

This temperature shifting enables us to construct the so-called master curve for a chosen reference temperature to express the response of the interlayer in a broader time or frequency domain. This procedure is called the time–temperature superposition principle (TTSP) and holds for the thermorheologically simple materials whose relaxation times are affected by the temperature in the same manner [26]. A guiding rule of thumb is that the TTSP may be used in the range of all tested temperatures as long as the measured data is shiftable to form a smooth master curve. Time–temperature shifting enables one to extend the range of relaxation times outside the range of experimental measurements. However, choosing the time instants or frequencies many decades far from the tested range may result in significant errors [26].

**Figure 2:**
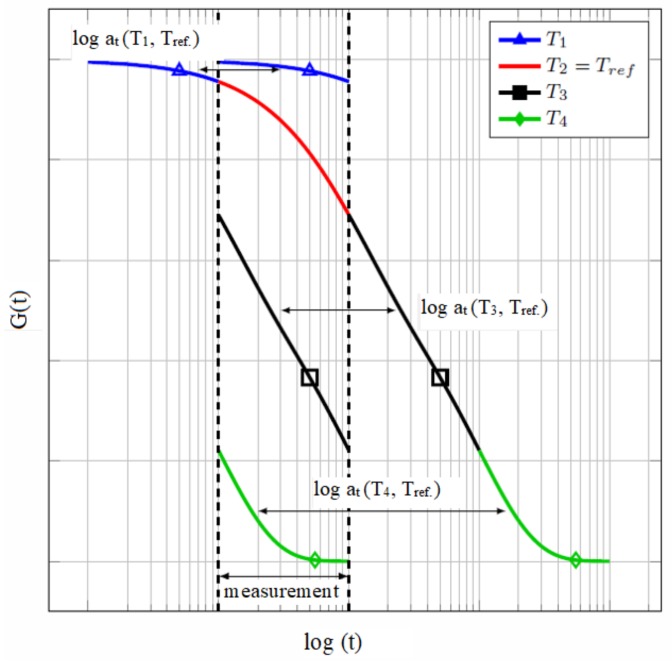
Time–temperature superposition principle master curve [29], $T_{1}<T_{2}<T_{3}<T_{4}$.

The shifting procedure is indicated in Figure 2. The shear stiffness modulus G(t) or parts of the complex dynamic modulus $G^{\prime }\left(\omega \right)$ and $G^{\prime \prime }\left(\omega \right)$ related to the measured temperature are horizontally shifted for the value of the shift factor $\log_{10}a_{T}$. The analytical expression of this factor can be evaluated through the William–Landel–Ferry equation (WLF) mentioned originally in [27] and adopted in the draft of the current European Standard [28] as

(8) $$\log_{10}a_{T}=-\frac{C_{1}\left(T-T_{0}\right)}{C_{2}+T-T_{0}},$$

with two free constants $C_{1}$ and $C_{2}$ and the reference temperature $T_{0}$, for which $a_{T}\left(T_{0}\right)=1$.

The use of the WLF equation is recommended in [26] for temperatures above the glass transition temperature $T_{g}$, whereas for lower temperatures $T\ll T_{g}$, the shift factor can be obtained through the Arrhenius activation energy equation [26]

(9) $$\log_{10}a_{T}=\frac{E_{a}}{2.303R}\left(\frac{1}{T}-\frac{1}{T_{g}}\right),$$

which is based on the activation energy of the tested polymer $E_{a}$ [J/mol] and the universal gas constant $R_{a}=8.3144621$ J/mol K.

**Figure 3:**
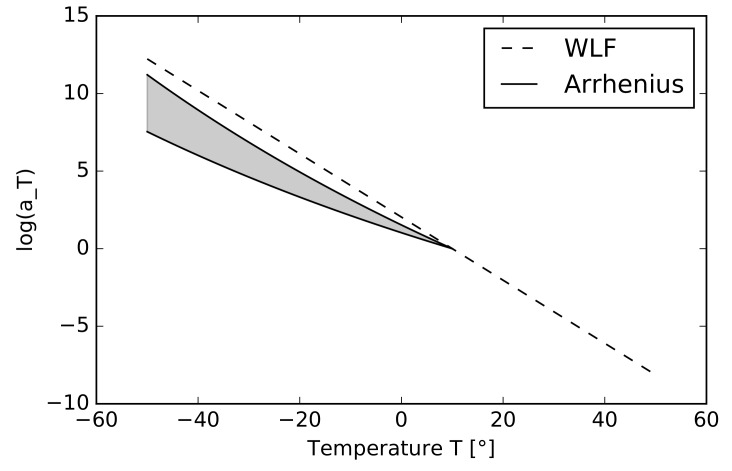
Comparison of PVB shift factors defined by the WLF equation and Arrhenius equations [26] for $T_{g}=10{\;}^{\circ }C$.

Note that adopting two different models for two ranges of temperature $T<T_{g}$ and $T>T_{g}$ becomes necessary only if the tested temperature is found far below the glass transition temperature [26]. For illustration, we plot in Figure 3 the variation of the shift factor $\log_{10}a_{T}$ as a function of temperature for the PVB interlayer, where the dashed line represents the WLF equation with the fitted parameters $C_{1}=8.635$ and $C_{2}=42.422$; see Section 4.3. Generally, the glass transition temperature and activation energy are wide range parameters. To support this claim, in [30] the glass transition region for PVB is declared to be between $10{\;}^{\circ }C$ and $20{\;}^{\circ }C$, and [31] declares the value of $T_{g}$ between $15{\;}^{\circ }C$ and $20{\;}^{\circ }C$. According to [32], the activation energy takes values between 152 and 226 kJ/mol. The highlighted area in Figure 3 represents the dispersion of the shift factor $\log_{10}a_{T}$ for this reported range of activation energies when setting $T_{g}=10{\;}^{\circ }C$ in Equation (). Note that for the lowest temperature $T=-10{\;}^{\circ }C$ considered in our experimental study, the difference of the shift parameter $\log_{10}a_{T}$ for the two limit values of activation energy is almost negligible, and that $E_{a}=226$ kJ/mol yields the value of $\log_{10}a_{T}$, almost identical to that provided by the WLF equation. Thus, exploiting only the WLF equation in the master curve fitting is sufficient.

### 2.4. Parameter Identification

The goal of the identification procedure is to fit the generalized Maxwell model enhanced by the time–temperature superposition principle to the experimental data in the form ${\left[{\bar{G}}_{r}^{\prime },{\bar{G}}_{r}^{\prime \prime },{\bar{\omega }}_{r},{\bar{T}}_{r}\right]}_{r=1}^{R}$, where *R* denotes the number of measurements. Thus, for every *r*-th pair of the prescribed angular frequency ${\bar{\omega }}_{r}$ and the prescribed temperature ${\bar{T}}_{r}$ we have available the measured values of the storage modulus ${\bar{G}}_{r}^{\prime }$ and the loss modulus ${\bar{G}}_{r}^{\prime \prime }$. Taking into account Equations (5), (6a), (6b) and (8), both the storage modulus $G^{\prime }$ and loss modulus $G^{\prime \prime }$ of the generalized Maxwell chain depend on the temperature *T*, the angular frequency ω, the shear moduli of the Maxwell units ${\left[G_{p}\right]}_{p=1}^{P}$, the shear modulus of the separate spring $G_{\infty }$, the shift factor parameters $C_{1}$, $C_{2}$, the reference temperature $T_{0}$ and the relaxation times of the Maxwell units ${\left[\theta_{p}\right]}_{p=1}^{P}$. The relaxation times have to be chosen so that they cover the range of the angular frequencies for which the storage modulus was measured. Having one or two Maxwell cells for each decade is a common choice for determining the number of Maxwell cells. Given Equation (2a), the storage modulus of the *p*-th Maxwell cell plotted in a logarithmic scale as a function of angular frequency ω increases in region $\omega <1/\theta_{p,0}$ and remains approximately constant for $\omega >1/\theta_{p,0}$. The storage modulus of the entire Prony series is thus a superposition of these approximately bilinear curves where the relaxation times determine their horizontal positions. Therefore, a homogeneous distribution of relaxation times $\theta_{p,0}$ in the logarithmic time scale, as also suggested in [33], is adopted.

Thus given the relaxation times ${\left[\theta_{p,0}\right]}_{p=1}^{P}$ and a fixed reference temperature $T_{0}$, we search for the values of ${\left[G_{p}\right]}_{p=1}^{P}$, $G_{\infty }$, $C_{1}$ and $C_{2}$ that minimize the objective function

(10) $$\begin{array}{c} F\left({\left[G_{p}\right]}_{p=1}^{P},G_{\infty },C_{1},C_{2}\right)=\sum_{r=1}^{R}\left({\left(G^{\prime }\left({\bar{\omega }}_{r},{\bar{T}}_{r}\right)-{\bar{G}}_{r}^{\prime }\right)}^{2}+{\left(G^{\prime \prime }\left({\bar{\omega }}_{r},{\bar{T}}_{r}\right)-{\bar{G}}_{r}^{\prime \prime }\right)}^{2}\right). \end{array}$$

The minimization procedure exploits the fact that the values of both $G^{\prime }$ and $G^{\prime \prime }$ obtained from (6a) and (6b) are linear in $G={\left\{G_{1}\cdots G_{P},G_{\infty }\right\}}^{T}$. Therefore, we can write the sum of squares (10) in the form

(11) $$\begin{array}{c} F\left(G,C_{1},C_{2}\right)={\left(X\left(C_{1},C_{2}\right)G-\bar{G}\right)}^{T}\left(X\left(C_{1},C_{2}\right)G-\bar{G}\right), \end{array}$$

where $\bar{G}={\left\{{\bar{G}}_{1}^{\prime }\cdots {\bar{G}}_{R}^{\prime },{\bar{G}}_{1}^{\prime \prime }\cdots {\bar{G}}_{R}^{\prime \prime }\right\}}^{T}$ stores the measured values of the storage and the loss moduli, and the components of the matrix

(12) $$\begin{array}{c} X\left(C_{1},C_{2}\right)=\left[\begin{array}{c} X^{\prime }\\ X^{\prime \prime } \end{array}\right], \end{array}$$

are provided by

(13) $$\begin{array}{c} X_{rp}^{\prime }=\frac{\theta_{p}^{2}\left({\bar{T}}_{r}\right){\bar{\omega }}_{r}^{2}}{1+\theta_{p}^{2}\left({\bar{T}}_{r}\right){\bar{\omega }}_{r}^{2}},\;X_{rp}^{\prime \prime }=\frac{\theta_{p}\left({\bar{T}}_{r}\right){\bar{\omega }}_{r}}{1+\theta_{p}^{2}\left({\bar{T}}_{r}\right){\bar{\omega }}_{r}^{2}} \end{array}$$

for r=1⋯R, p=1⋯P. Values in the last, i.e., (P+1)-th column of $X^{\prime }$ and $X^{\prime \prime }$, are equal to one and zero, respectively. Note that the dependence of X on the shift factor parameters $C_{1}$ and $C_{2}$ follows from Equation (8). According to the ordinary least squares method, the optimal values of G are

(14) $$\begin{array}{c} \hat{G}\left(C_{1},C_{2}\right)={\left(X^{T}X\right)}^{-1}X^{T}\bar{G}. \end{array}$$

Substituting these optimal values back to Equation (11) provides the objective function in the form

(15) $$\begin{array}{c} F\left(C_{1},C_{2}\right)={\left(X{\left(X^{T}X\right)}^{-1}X^{T}\bar{G}-\bar{G}\right)}^{T}\left(X{\left(X^{T}X\right)}^{-1}X^{T}\bar{G}-\bar{G}\right), \end{array}$$

so it depends on the shift factor parameters only. To find the optimal values $C_{1}$ and $C_{2}$ that minimize function $F(C_{1},C_{2})$, the Nelder–Mead method [34] was employed. The choice of this method over other minimization techniques was mainly motivated by the fact that it does not require the gradient of the minimized function to be known.

## 3. Experimental Methods

The mechanical tests to determine the properties of polymeric interlayers can be performed on small-size samples of a single interlayer [2,35], on a small laminated glass specimen [8,36], or on large specimens [9,37]. Using large-scale experiments, the size and edge effects are minimized [38]. On the other hand, thermal control is generally more difficult in comparison to small-size specimens.

According to the loading scenario, the methods can be classified as (quasi)-static monotonic tests, creep or stress-relaxation experiments, and dynamic tests [39]. In some studies, the identification is based on the probabilistic Bayesian approach [40]. The material data for the polymer can also be deduced from a combination of these tests using an interconversion between different deformation modes [15]. The draft standard [28] is devoted to the determination of mechanical properties and provides a few testing methods related to different types of interlayers.

Monotonic tests are useful to get the first insight into the behavior of an interlayer. The rheological behavior under long-term loading is analyzed using creep or relaxation tests as well as dynamic tests. For impact or explosion analyses, dynamic tests are applied to study the properties in the frequency range above 100 Hz [36].

Periodic experiments such as cyclic tensile, shear, or torsion tests represent a large group of commonly used techniques. Dynamic mechanical analyses based on the cyclic tensile test on a pure foil strip can be found in many studies on laminated glass, e.g., [2,10,35]. Subjecting the interlayer to a shear stress state such as in [36] or [8], the shear modulus is directly obtained from the test without the need for any conversion through the Young modulus using an estimated or experimentally determined Poisson ratio or bulk modulus. Therefore, the shear or torsion tests seem to be more reliable techniques avoiding the conversion from tests in tension. The single-lap shear tests, Section 3.1, and the cyclic torsional tests, Section 3.2, of small three-layer samples of laminated glass are adopted in this study to identify the complex shear modulus with its real and imaginary part.

### 3.1. Dynamic Single-Lap Shear Test

#### 3.1.1. Test Setup

**Figure 4:**
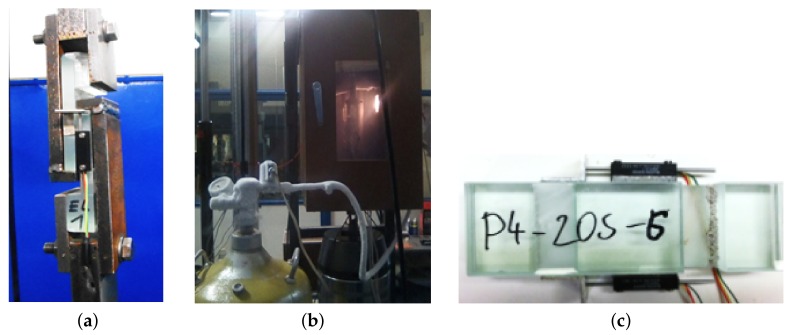
(**a**) MTS machine metal jaws of a single-lap shear test, (**b**) climatic chamber for dynamic mechanical thermal analysis (DMTA) in shear and Dewar vessel, (**c**) specimen with potentiometric linear transducers.

A dynamic mechanical thermal analysis (DMTA) in a single-lap shear test was carried out first using the MTS 500B testing device manufactured by MTS Systems Corporation, Eden Prairie, MN, USA, and equiped with a climatic chamber as seen in Figure 4a,b. To apply low temperatures in the chamber during testing, the liquid nitrogen stored in a Dewar vessel was blown directly into the chamber. The temperature of the glass surface was measured by two Pt 100 temperature sensors attached to both sides of the specimen. The potentiometric linear transducers MMR 1011 displayed in Figure 4c were used to measure a relative slippage of the glass plies *u* in [mm] in the direction of the acting force.

**Figure 5:**
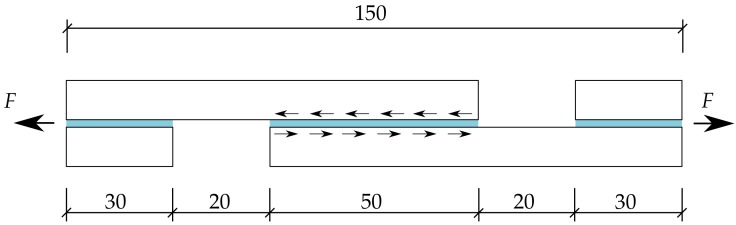
Nominal dimensions of the testing specimen in [mm].

**Figure 6:**
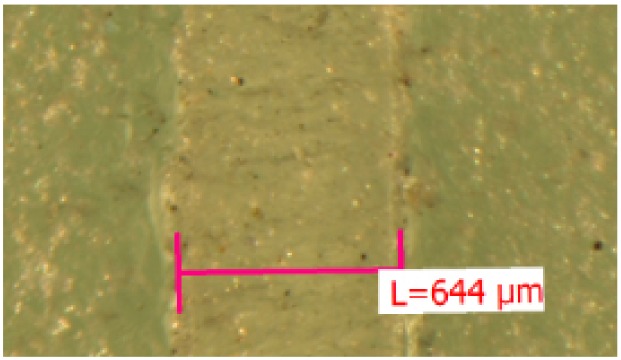
Measured EVA interlayer representative thickness.

Test specimens, schematically plotted in Figure 5, were made of 2 annealed float glass panes bonded in an autoclave with the transparent PVB and EVA interlayers with a shear area of 50×50 mm. In total, six PVB and eight EVA specimens were prepared. Since it was needed for the test evaluation, the thickness of the interlayer for each specimen was determined from the measurements at three points along the shear area of every specimen using a microscope. An illustrative example is plotted in Figure 6. The average values of the thickness of PVB and EVA specimens, used in the evaluation of results, were found to be equal to 1.49 mm and 0.64 mm, respectively. The nominal thickness of PVB and EVA was declared by the manufacturer to be 1.52 mm and 0.76 mm, respectively. Thus, the lamination process caused a slight transverse compression of the tested interlayers.

**Figure 7:**
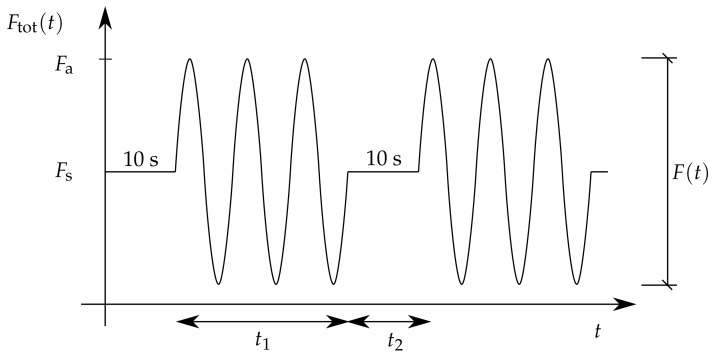
Measured force in time during the test; during the time interval $t_{1}$, the sample is displacement controlled, and during the time interval $t_{2}$, the sample is force controlled.

As seen in Figure 4a, the specimens were fixed in between two metal jaws of the testing device and loaded dynamically following the course displayed in Figure 7, where $F_{s}$ refers to the tensile prestressing force between each cycle set to 1.2–1.5 kN depending on the tested interlayer and temperature. These values assured reliable fastening of the specimen in the metal jaws during cycling. The duration of the prestressing force between cycles was set to 10 s.

Individual loading cycles were displacement controlled. The amplitude of the MTS loading cylinder displacement during cycling $u_{\max}$ was set in the range of 0.17–0.2 mm such that the maximum force $F_{a}$ reached in the loading cycle would stay below 3.0 kN to avoid the specimen’s failure. The temperature and loading frequency were changed during the tests. The range of testing frequencies was set to 0.05–4.95 Hz and the frequency step between cycles was set to 0.05 Hz. The testing temperatures were assumed in the range of $-5{\;}^{\circ }$C to $+40{\;}^{\circ }$C for PVB and $-10{\;}^{\circ }$C to $+50{\;}^{\circ }$C for EVA specimens, with the step set to $5{\;}^{\circ }$C. Both the temperature and prestressing force, as well as the amplitude of the cylinder displacement, were kept constant during all 99 cycles.

The prestressing force and the applied displacement of the loading cylinder resulted in the total shear strain and the corresponding shear stress of the interlayer given by

(16) $$\begin{array}{c} \gamma_{\mathrm{tot}}\left(t\right)=\gamma \left(t\right)+\gamma_{s}, \end{array}$$

(17) $$\begin{array}{c} \tau_{\mathrm{tot}}\left(t\right)=\tau \left(t\right)+\tau_{s}, \end{array}$$

where γ(t) and τ(t) represent the dynamic shear strain and stress, respectively. The static shear strain and stress components $\gamma_{s}$ and $\tau_{s}$ are associated with the prestressing force $F_{s}$.

#### 3.1.2. Method of Results Evaluation

Given the geometry of the tested specimen in Figure 5, the shear strain of the interlayer was found from a simple definition of the engineering shear strain as

(18) $$\gamma \left(t\right)=\frac{u(t)}{h_{f}},$$

where u(t) is the measured relative slip between the two glass plies, and $h_{f}$ is the average thickness of the interlayer. In each cycle, the dynamic shear strain of the interlayer was controlled by the formula

(19) $$\gamma \left(t\right)=\gamma_{\max}\sin\omega t,$$

where ω is the loading angular frequency, *t* is the instantaneous time in each cycle, and $\gamma_{\max}$ is the amplitude of the dynamic shear strain of the interlayer derived from the amplitude of the MTS loading cylinder displacement $u_{\max}$. The corresponding dynamic shear stress follows from

(20) τ(t)=F(t)/A,

where *A* is the shear area of the interlayer, and F(t) is the measured force. Since polymeric interlayers are viscoelastic materials, a certain phase shift δ between the strain and stress occurs during the loading cycle, which is attributed to the dissipation of mechanical energy [41] and expresses the rate of material viscosity. When δ=0, then the material is elastic; when δ=π/2, then the material is purely viscous. The measured force and the applied loading cylinder displacement were out of phase during cycling for both tested interlayers, thus proving their viscoelastic response. Therefore, the dynamic shear stress can also be expressed as

(21) $$\tau \left(t\right)=\tau_{\max}\sin\left(\omega t+\delta \right).$$

**Figure 8:**
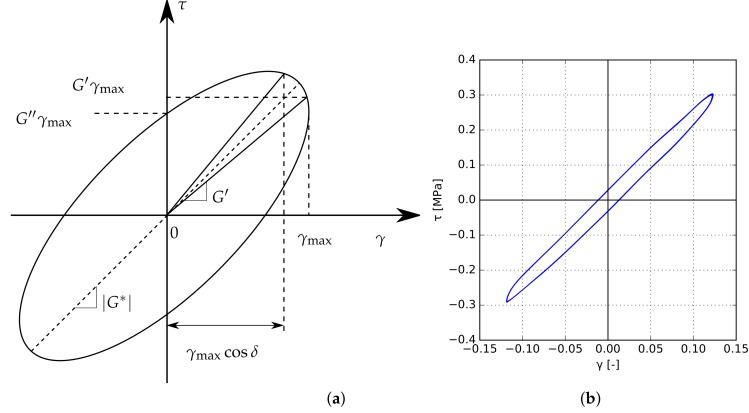
Viscoelastic loop of dynamic stress–strain relation in a loading cycle: (**a**) illustrative scheme showing all important points, (**b**) example of EVA stress–strain relation observed during cycling.

The dynamic stress–strain dependence in each loading cycle may be graphically displayed through the viscoelastic loop plotted in Figure 8a. Figure 8b illustrates, as an example, one such loop obtained experimentally for an EVA specimen.

The viscoelastic loop shows some important points that can be adopted in the evaluation of the time- and temperature-dependent shear modulus G(t,T). The slope of the loop symmetry axis indicates the absolute value of the dynamic complex shear modulus $|G^{*}|$. The slope of the line passing through the origin of the coordinate system and the point at which the maximum shear deformation is achieved indicates the value of the shear storage modulus $G^{\prime }$, which is directly proportional to the average energy stored in each loading cycle [22]. The intersection of the hysteresis loop with the shear stress axis allows for the determination of the shear loss modulus $G^{\prime \prime }$.

To analytically express the shear stress response τ(t) of the interlayer to the sinusoidal dynamic shear strain input according to Equation (19), the Boltzmann superposition principle is applied. The final expression can be conveniently written in terms of the storage and loss moduli $G^{\prime }$ and $G^{\prime \prime }$ [41] as

(22) $$\tau \left(t\right)=G^{\prime }\gamma_{\max}\sin\omega t+G^{\prime \prime }\gamma_{\max}\cos\omega t.$$

When ωt=π/2, $\gamma_{\max}$ is achieved, and then $\tau \left(t=\pi /\left(2\omega \right)\right)=G^{\prime }\gamma_{\max}$. On the other hand, for time t=0 it holds γ=0, therefore $\tau \left(0\right)=G^{\prime \prime }\gamma_{\max}$. Exploiting these important points shown on the viscoelastic loop in Figure 8a, the complex shear modulus with its real and imaginary parts was evaluated from the experimental loops, such as the one in Figure 8b, for each loading cycle. Having determined these moduli for the tested temperatures and frequencies, the Prony series can further be identified following the calibration procedure from Section 2.4 to obtain the shear stiffness modulus G(t) in the time domain.

### 3.2. Dynamic Torsion Tests

#### 3.2.1. Test Set-Up

**Figure 9:**
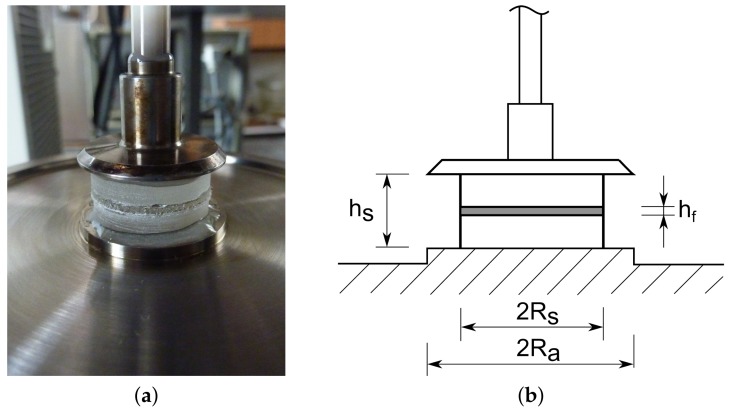
Rheometer device: (**a**) mounted sample, (**b**) geometrical details.

The second approach to the experimental investigation of the viscoelastic response of polymeric interlayers follows the work in [36] and adopts a dynamic shear rheometer HAAKE MARS manufactured by Thermo Fisher Scientific, USA, see Figure 9. As instrumental software HAAKE RheoWin 4.30.0017 was used. This device operates on the plate–plate shear principle and allows for stress as well as strain loading conditions applied either in static (simple rotation) or dynamic (oscillatory) mode under the prescribed temperature.

The measurements were performed on small, layered, cylindrical samples having a diameter of 20 mm and thickness of 5 + $h_{f}$ + 5 mm. To ensure that the oscillatory loading was taken by the interlayer only, a stiff epoxy glue had to be applied when mounting the specimen on the two plates. The top rotating plate was used to prescribe the oscillatory loading. The bottom plate was fixed and served also as a heat source to generate the required testing temperature by thermal contact conduction. The steady-state thermal conditions were ensured by covering the whole assembly by a Teflon case.

The specimens were drilled directly from a laminated glass with two-ply PVB and EVA interlayers; see Figure 9a. The average thickness $h_{f}$ of all tested specimens (4 PVB and 7 EVA samples) was again measured with micrometer to arrive at 0.73 mm and 0.8 mm for the PVB and EVA interlayers, respectively.

Unlike a single-lap shear test, the specimens were loaded in a stress-controlled regime by prescribing the torsional shear stress. The oscillatory tests were performed for the following set of frequencies: 0.001 Hz, 0.01 Hz, 0.05 Hz, 0.1 Hz, 0.5 Hz, 1 Hz, 5 Hz, 10 Hz, 20 Hz, 30 Hz, 40 Hz, 50 Hz. The assumed temperatures ranged from $10{\;}^{\circ }C$ to $60{\;}^{\circ }C$, with a $10{\;}^{\circ }C$ step for both interlayers.

#### 3.2.2. Method of Results Evaluation

The rheometer directly provides the frequency-dependent variation of the storage and loss moduli for a given temperature assuming constant strain distribution across the sample height $h_{s}$; recall Figure 9b. Moreover, the known adapter radius $R_{a}$ is used to transform the prescribed torsional shear stress to the torsional moment. This is an adequate premise for the asphalt mixture for which the rheometer was originally constructed, but it is no longer sufficient for the laminated glass samples. Thus, the experimentally provided complex moduli $G_{s}^{*}\left(\omega \right)$ must be properly adjusted. As suggested in [36], it is reasonable to assume that the entire torsion is concentrated in the polymeric interlayer with height $h_{f}$ and radius $R_{s}$ to get the complex modulus $G_{f}^{*}\left(\omega \right)$ of the interlayer as [36,42]

(23) $$G_{f}^{*}\left(\omega \right)=G_{s}^{*}\left(\omega \right)\frac{R_{a}^{4}}{R_{s}^{4}}\frac{h_{f}}{h_{s}}.$$

As already mentioned in Section 3.1.2, the derived moduli $G_{f,r}^{*}\left({\bar{\omega }}_{r},{\bar{T}}_{r}\right)$ can now be introduced into the identification procedure outlined in Section 2.4 to calibrate the Maxwell chain model.

## 4. Results

### 4.1. Linearity Validation

Prior to performing any comparative study of these experimental approaches, we first examine the legitimacy of the application of the theory of linear viscoelasticity in describing the behavior of the adopted polymeric interlayers.

In the theory of linear viscoelasticity, the shear modulus of polymeric interlayers *G* depends only on the load duration and temperature, and the stress–strain relationship in a relaxation test is linear at a certain time *t*. Splitting the relaxation function $G\left(t\right)=G_{\infty }+\hat{G}\left(t\right)$, where $G_{\infty }=\lim_{t\to \infty }G\left(t\right)$ is the equilibrium modulus, allows us to write the shear storage and loss moduli with the help of variable $\psi =t-t^{\prime }$ as

(24) $$\begin{array}{ccc} G^{\prime }\left(\omega \right) & = & G_{\infty }+\omega \int_{0}^{\infty }\hat{G}\left(\psi \right)\sin\omega \psi d\psi , \end{array}$$

(25) $$\begin{array}{ccc} G^{\prime \prime }\left(\omega \right) & = & \omega \int_{0}^{\infty }\hat{G}\left(\psi \right)\cos\omega \psi d\psi , \end{array}$$

further suggesting the shear modulus *G* be only dependent on time, and the storage $G^{\prime }$ and loss $G^{\prime \prime }$ be only dependent on frequency. Note that Equations (24) and (25) follow from the Boltzmann superposition principle applicable to linear viscoelasticity. The validity of the assumed linear viscoelasticity will be now verified for both types of experiments in a sequel.

We begin with the single-lap shear test, (Section 3.1), subjecting the specimen to oscillatory loading according to Equations (19) and (20). The amplitudes of displacement $u_{\max}$ varied from 0.03 mm to 0.25 mm with the step of 0.01 mm at a constant frequency of 1 Hz and constant temperatures $T=40{\;}^{\circ }$C (PVB) and $T=50{\;}^{\circ }$C (EVA). The prestressing force was set to 1.2 kN and 1.4 kN for the PVB and EVA interlayers, respectively. Note that the temperatures are the maximum ones used in the actual experimental campaign for which the potential stress–strain nonlinearity might be expected [43].

**Figure 10:**
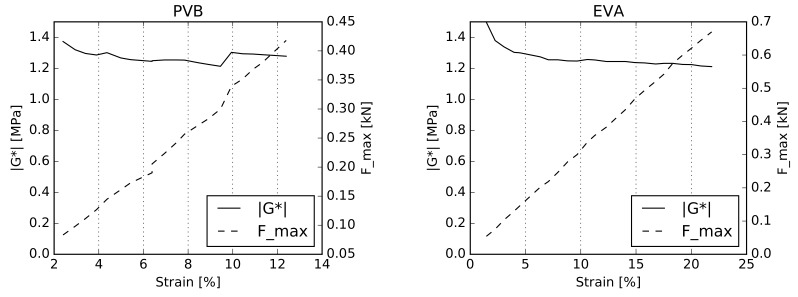
PVB and EVA linearity check for the selected amplitude sweep at a frequency of 1 Hz in the single-lap shear test.

The relation between the dynamic complex shear modulus $G^{*}$ and the amplitude of the dynamic engineering shear strain of the interlayer $\gamma_{\max}$ as well as the maximum measured dynamic force $F_{\max}$ are shown in Figure Figure 10 for both tested materials. The dynamic complex shear modulus in both cases was found to be in the range of 1.2–1.4 MPa, which is almost constant in the interval of the tested displacement amplitudes $u_{\max}$. Note also that the measured maximum dynamic force $F_{\max}$ depends linearly on the applied strain. These results, therefore, allow for the assumption of the stress–strain linearity at a certain time.

**Figure 11:**
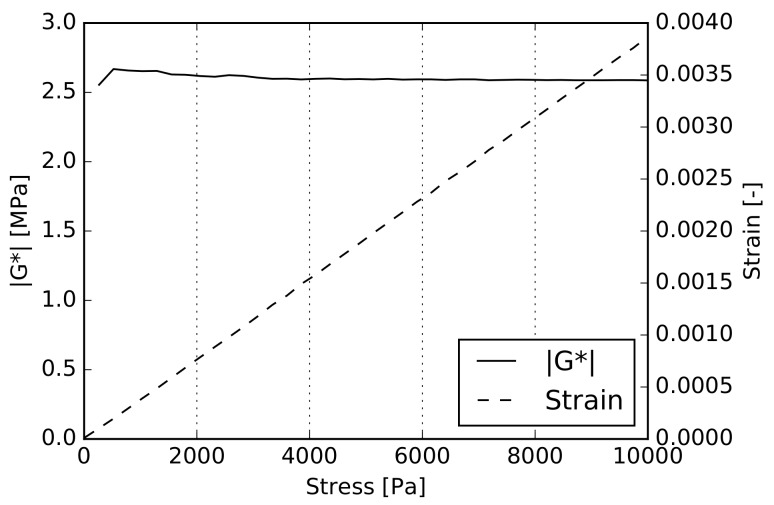
PVB linearity test at $60{\;}^{\circ }$C for the dynamic torsion test.

In the next step, the linearity check was also performed for the dynamic torsion test, limiting our attention to the PVB interlayer only. The experiment was carried out again at the maximum testing temperature $T=60{\;}^{\circ }$C. The sample was loaded with a uniformly increasing torsional moment while recording the corresponding complex modulus $G^{*}$, adjusted according to Equation (23). The result is shown in Figure 11, indicating, again, an almost constant value of $|G^{*}|$ with increasing stress and linearity of the stress–strain relation.

Both tests thus confirmed the applicability of the viscoelastic Maxwell chain model in potential numerical simulations.

### 4.2. Comparison of Obtained Results

This section serves to compare the results provided by the presented experimental approaches.

**Figure 12:**
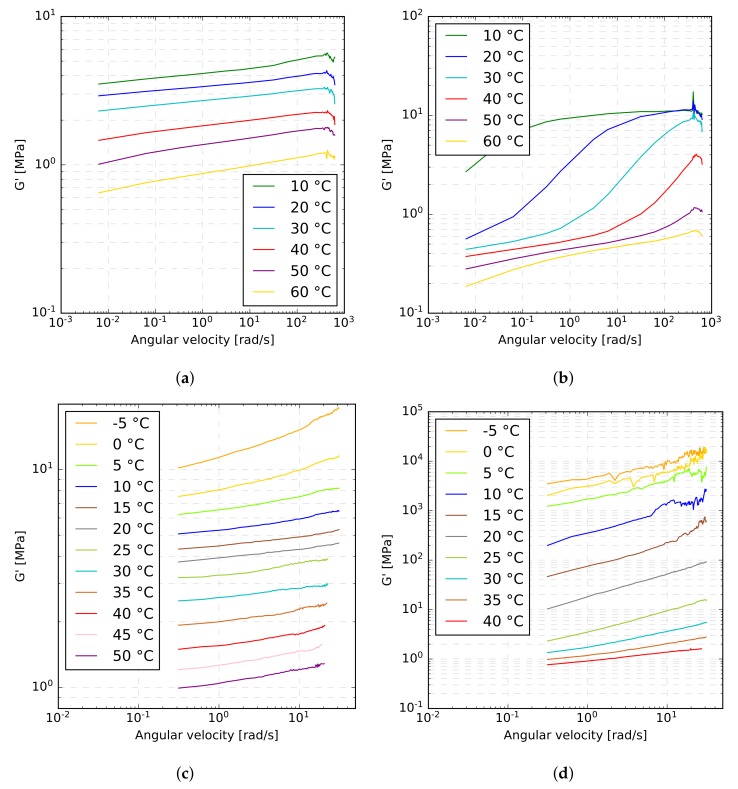
Storage modulus–frequency relations for a selected EVA (**a**,**c**) and PVB (**b**,**d**) specimen: (**a**,**b**) dynamic torsion test, (**c**,**d**) single-lap shear test.

**Figure 13:**
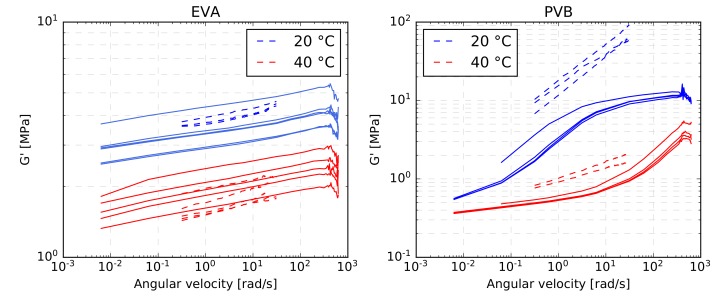
Comparison of all EVA and PVB shear and torsion measurements for temperatures of $20{\;}^{\circ }$C and $40{\;}^{\circ }$C (solid lines correspond to the dynamic torsion test, dashed lines to the single-lap shear test).

Figure 12 shows the variation of the storage modulus $G^{\prime }\left(\omega \right)$ for both types of interlayers derived from one particular sample in each type of experiment. It is seen in Figure 12a,c that the curves belonging to the EVA interlayer are of similar shape with comparable slopes for both experimental methods. However, such an agreement was not observed for the PVB interlayer, as evident from Figure 12b,d, particularly for lower temperatures, for which the PVB interlayer experienced a considerably stiffer response in the single-lap shear test in comparison to the dynamic torsion test. A similar conclusion can be drawn from Figure 13, where all measurements at temperatures of $20{\;}^{\circ }$C and $40{\;}^{\circ }$C are plotted together. Figure 13 shows relatively high variation between samples for both experimental methods. In particular, the measured values of the storage modulus of the EVA interlayer differ for some samples by almost 50%, but there is no clear difference for the two experimental methods. This, however, does not hold for PVB material, where the single-lap shear test gives higher values of storage modulus than the dynamic torsion test. The discrepancy is more pronounced for lower temperatures and higher angular frequencies, where the response of the PVB material is stiffer. The exact reason for this deviations is unknown to the authors, but it might be connected to a lower accuracy of one of the methods when dealing with stiffer material, resulting in small measured displacements or rotations.

### 4.3. Identification of the Maxwell Model

Several master curves were constructed for both types of interlayers using the identification procedure developed in Section 2.4. While it was generally possible to account for both storage and loss moduli (recall Equation (10)), the frequency-dependent distributions of loss moduli provided by single-lap shear tests were too noisy to allow for their application in the identification procedure. On the other hand, the objective was to employ both types of experiments, treating them on the same footing. This is why only the data associated with the storage modulus were eventually used.

**Figure 14:**
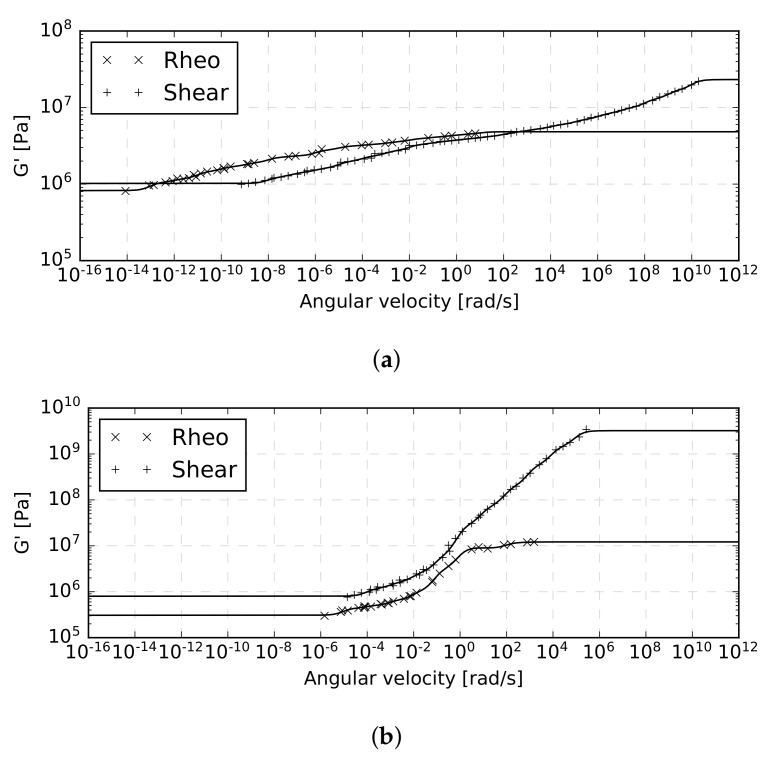
Representative master curves constructed at $20{\;}^{\circ }$C with both experimental methods: (**a**) EVA interlayer, (**b**) PVB interlayer; rheo indicates the dynamic torsion test, shear indicates the single-lap shear test.

An illustrative example of storage modulus master curves constructed at $20{\;}^{\circ }$C for a representative specimen with either a PVB or EVA interlayer is plotted in Figure 14. It is obvious that for one specimen tested by one experimental method, the fitting algorithm is able to construct the resulting master curve with good accuracy.

Notice that in the area of the plotted extreme angular velocities, the experimental data are missing. There, the storage modulus is covered by the Maxwell chain model only, where it delivers constant values of this modulus. Thus, to improve the range of applicability of the presented model, the range of testing temperatures or frequencies would have to be extended, calling also for subsequent recalibration of the model.

However, given the results in Figure 12 and Figure 13, it is not clear what data set should be used to arrive at the most representative master curve. A distinct variability in the obtained results is observed not only for the two experimental methods (Figure 12 and Figure 13), but also for individual specimens tested at the same temperature by the same method (Figure 13). Because of this, the final master curve of the shear storage modulus at $20{\;}^{\circ }$C for each interlayer was constructed from all available data sets, i.e., data from all measurements of storage moduli provided by both experimental methods were considered simultaneously.

**Figure 15:**
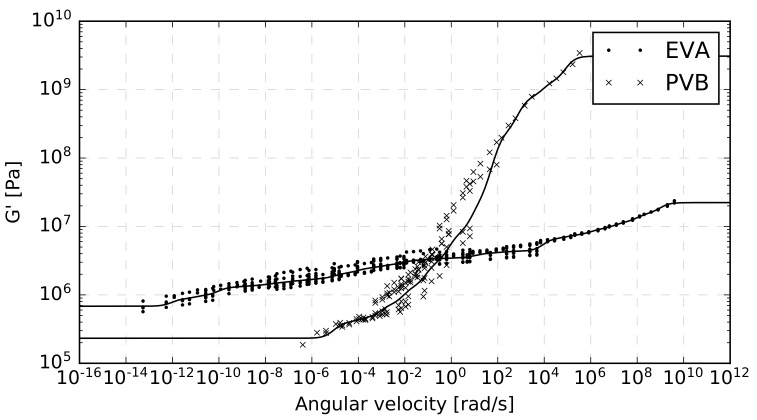
Master curves fitted to all experimental data at $20{\;}^{\circ }$C for the EVA and PVB interlayers.

These master curves are presented in Figure 15. The points correspond to the measured data, and the curves represent the response of the Maxwell model. It is clearly visible that due to the variability of the measured data, the deviation of the optimal master curve from the data points is much higher than in the cases where only specific data series were considered; compare Figure 14 and Figure 15. In particular, the sum of squared relative errors is 2.63 for EVA and 4.51 for PVB when all measured data provided by both experimental methods is used. Compare this to values of 0.15 (single-lap shear) and 0.12 (dynamic torsion) for EVA curves in Figure 14a and 0.44 (single-lap shear) and 0.15 (dynamic torsion) for PVB curves in Figure 14b. The data points are sparse in area, with the angular velocity above 10${}^{4}$ rad/s (EVA) and above 10${}^{2}$ rad/s (PVB). This area corresponds to low temperatures, for which only single-lap shear tests were performed. The experimental shear modulus in Figure 15 tends to decrease (increase) within low (high) angular velocities. This is in agreement with the physical nature of the model. In high velocities, the viscous part of the spring-damper element is stifled, and the stiffness of the model approaches the stiffness of all springs. On the other hand, when the load is applied slowly, the viscosity prevails, and the stiffness is dramatically reduced. The values of the PVB shear storage modulus are much higher in comparison to the EVA modulus for angular velocities above 1 rad/s. For lower velocities, the situation is inverse. The PVB master curve has a steeper slope than EVA. A noteworthy increase of $G^{\prime }$ for PVB is evident for angular frequencies between 1 and 10${}^{2}$ rad/s. It may thus be expected that PVB attains a more pronounced decrease in stiffness for a short-term loading than EVA.

Fitted parameters of the generalized Maxwell model as well as the additional WLF constants at $20{\;}^{\circ }$C for the time–temperature shifting are summarized in Table 1 for both interlayers. If a sudden strain in a relaxation test is applied, the corresponding shear stiffness tends to the sum of the stiffness of all springs. The PVB initial shear stiffness is $G_{0,\mathrm{PVB}}=$ 3085 MPa, and the EVA initial shear stiffness is $G_{0,\mathrm{EVA}}=22.3$ MPa. To obtain the corresponding shear stiffness modulus in the time domain $G(T=20{\;}^{\circ }C,t)$, it is necessary to use Equation (7) with the fitted Maxwell input parameters. Supposing the thermorheological simplicity of both interlayers, the fitted relaxation times $\left[\theta_{p}\left(T_{0}\right)\right]$ can be extrapolated to other tested temperatures $\left[\theta_{p}\left(T\right)\right]$ through Equation (5) to obtain a new set of storage $G^{\prime }\left(T,t\right)$ and loss $G^{\prime \prime }\left(T,t\right)$ moduli using WLF constants $C_{1}$ and $C_{2}$ in Table 1. The corresponding shear stiffness modulus G(t,T) can then be also calculated using Equation (7). The shear stiffness at long relaxation times is expressed through the equilibrium modulus. Values of this modulus are negligible, with $G_{\infty }<0.7$ MPa for both interlayers.

## 5. Conclusions

The aim of our study was to identify the material parameters of the generalized Maxwell chain model for two types of interlayers such as PVB-based TROSIFOL^®^ BG R20 and EVA-based EVALAM^®^ 80-120, enabling the description of their time- and temperature–dependent behavior. To this purpose, sets of small-scale cyclic single-lap shear tests in an MTS loading device and torsional tests in a rheometer were performed for the range of temperatures from $-10{\;}^{\circ }$C to $+60{\;}^{\circ }$C and the range of frequencies from 0.001 Hz to 50 Hz .

Having experimentally verified the applicability of linear viscoelasticity to these materials, the measured values of the storage and loss moduli allowed us to construct master curves of the shear storage modulus and to identify the material parameters of the generalized Maxwell chain model. Time–temperature shifting through the shift coefficient $a_{T}\left(T\right)$ based on the widely recognized WLF equation enabled us to extend the range of experimental data and test frequencies outside the range of experimental measurements. The Prony series were optimized using a combination of the linear least square method and the Nelder–Mead method to fit the measured data. The main advantage of using the generalized Maxwell model together with the WLF equation for the temperature effect is that it allows for expressing not only the storage and loss modulus in the frequency domain but also the relaxation function G(T;t) in the time domain. Its application in the dynamic finite element analysis is therefore straightforward.

The comparison of master curves of both materials shows that the PVB curve is steeper and attains more than one hundred times higher values of the shear storage modulus at extremely high frequencies than EVA. On the other hand, in low frequencies, EVA attains almost four times higher values of this modulus. In particular, the EVA master curve for the reference temperature of $20{\;}^{\circ }$C ranges from $G^{\prime }=7\times 10^{5}$ Pa at $\omega =10^{-13}$ rad/s to $G^{\prime }=3\times 10^{7}$ Pa at $\omega =10^{8}$ rad/s, while the master curve for PVB material ranges from $G^{\prime }=2\times 10^{5}$ Pa at $\omega =10^{-6}$ rad/s to $G^{\prime }=3\times 10^{9}$ Pa at $\omega =10^{5}$ rad/s. Although the PVB showed a generally stiffer response compared to EVA, this difference disappears for temperatures of $40{\;}^{\circ }$C and higher (recall Figure 12).

Nevertheless, the fact that the measured values of storage shear modulus for a given frequency and temperature differ not only from one experimental method to the other (see Figure 14) but also from specimen to specimen (see Figure 13) indicates some uncertainty in the obtained parameters of the Maxwell model itself. Thus, the aim of the next research is to validate the parameters of a viscoelastic model based on the experimentally measured and numerically predicted response of large-scale samples.

## Figures and Tables

**Table 1:** Parameters for the generalized Maxwell model for PVB (TROSIFOL^®^ BG R20) and EVA interlayers (EVALAM 80-120).

**Polymer**					**PVB**	**EVA**	
Long-term shear modulus					232.26	682.18	kPa
Reference temperature				$T_{0}$	20	20	${}^{\circ }C$
Parameters				$C_{1}$	8.635	339.102	–
				$C_{2}$	42.422	1185.816	${}^{\circ }C$
		**PVB**	**EVA**			**PVB**	**EVA**
p	***θ_p_***	***G_p_***	***G_p_***	p	***θ_p_***	***G_p_***	***G_p_***
	**[s]**	**[kPa]**	**[kPa]**		**[s]**	**[kPa]**	**[kPa]**
1	$10^{-9}$	–	6933.9	12	$10^{2}$	587.2	445.1
2	$10^{-8}$	–	3898.6	13	$10^{3}$	258.0	300.1
3	$10^{-7}$	–	2289.2	14	$10^{4}$	63.8	401.6
4	$10^{-6}$	–	1672.7	15	$10^{5}$	168.4	348.1
5	$10^{-5}$	1,782,124.2	761.6	16	$10^{6}$	–	111.6
6	$10^{-4}$	519,208.7	2401.0	17	$10^{7}$	–	127.2
7	$10^{-3}$	546,176.8	65.2	18	$10^{8}$	–	137.8
8	$10^{-2}$	216,893.2	248.0	19	$10^{9}$	–	50.5
9	$10^{-1}$	13,618.3	575.6	20	$10^{10}$	–	322.9
10	$10^{0}$	4988.3	56.3	21	$10^{11}$	–	100.0
11	$10^{1}$	1663.8	188.6	22	$10^{12}$	–	199.9
